# Omic research in termites: an overview and a roadmap

**DOI:** 10.3389/fgene.2015.00076

**Published:** 2015-03-13

**Authors:** Michael E. Scharf

**Affiliations:** Department of Entomology, Purdue University, West Lafayette, INUSA

**Keywords:** holobiome, digestome, sociogenomics, symbiosis, metabolomics, DNA methylation, sociobiology, socioevolution

## Abstract

Many recent breakthroughs in our understanding of termite biology have been facilitated by “omics” research. Omic science seeks to collectively catalog, quantify, and characterize pools of biological molecules that translate into structure, function, and life processes of an organism. Biological molecules in this context include genomic DNA, messenger RNA, proteins, and other biochemicals. Other permutations of omics that apply to termites include sociogenomics, which seeks to define social life in molecular terms (e.g., behavior, sociality, physiology, symbiosis, etc.) and digestomics, which seeks to define the collective pool of host and symbiont genes that collaborate to achieve high-efficiency lignocellulose digestion in the termite gut. This review covers a wide spectrum of termite omic studies from the past 15 years. Topics covered include a summary of terminology, the various kinds of omic efforts that have been undertaken, what has been revealed, and to a degree, what the results mean. Although recent omic efforts have contributed to a better understanding of many facets of termite and symbiont biology, and have created important new resources for many species, significant knowledge gaps still remain. Crossing these gaps can best be done by applying new omic resources within multi-dimensional (i.e., functional, translational, and applied) research programs.

## Introduction

### Overview and Terminology

**FIGURE 1:**
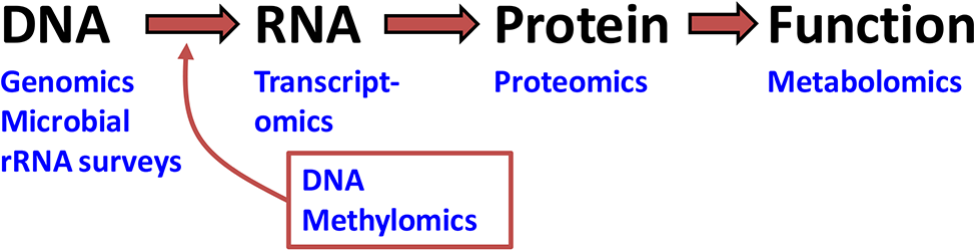
**The continuum of biological organization and function addressed by omic research**. The three bio-molecules listed (DNA, RNA, and protein) constitute the Central Dogma of Biology. Omic approaches that target these molecules can at best infer function. Proving function requires metabolomics and other functional or translational approaches not covered in this review (Scharf, 2015).

In a broad sense, the underlying goals of omic^1^ science are to catalog, quantify, and characterize pools of biological molecules that translate into structure, function, and life processes of an organism or environment. The types of biological molecules receiving focus in omics^2^ include genomic DNA, messenger RNA (mRNA), protein, and metabolites (**Figure 1**). DNA, mRNA, and protein are respectively the foci of genomics, transcriptomics, methylomics, and proteomics. Genomics, methylomics, and transcriptomics rely on nucleic acid sequencing, whereas proteomics utilizes peptide sequencing procedures. By contrast, metabolomics is rooted more in analytical chemistry and focuses on biochemicals, metabolites, or pathways. Another relevant omic approach is the cataloging of bacterial and protist symbionts using high-throughput 16S and 18S rRNA sequencing.

Termite omic research has focused on the host termite, individual gut microbial symbionts or entire populations of gut microbes. In the latter case, these “meta” analyses focusing broadly on collective microbiota occurring in the gut microenvironment have been popular, particularly with microbiologists specializing in termite intestinal microbiology. Although it presents significant bioinformatic challenges, a more inclusive approach that considers host and symbionts together as a single functional unit is the best approach for appreciating the full functional capacity of termites. A fundamental advantage of omic research over more traditional organismal research is that it enables direct mechanistic insights into termite and symbiont physiology and biochemistry. The use of omic technologies has led to new insights into behavior, social structure, digestion, and host-symbiont/symbiont–symbiont interactions, and many other aspects of termite biology. However, also as addressed throughout this review, omic science has limits for being able to define biological function.

### Termite Symbiosis and the Holobiont Concept

**FIGURE 2:**
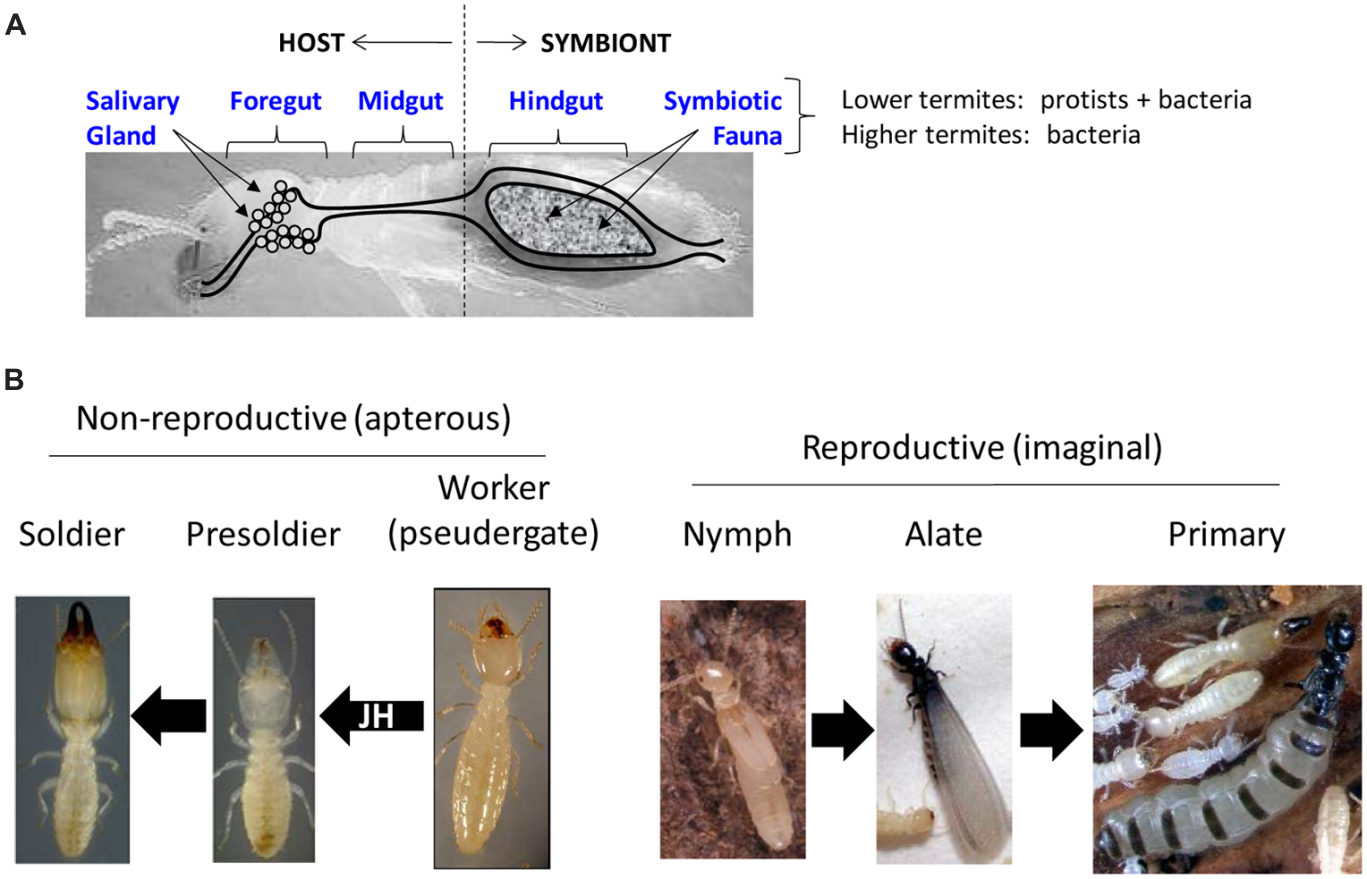
**Fundamental ideas behind digestomic and sociogenomic research in termites. (A)** Key components associated with termite digestomes and digestomic research. Different gut regions have been studied in an attempt to dissect host and symbiont contributions to digestion. An important distinction between lower and higher termites is the presence of protist and bacterial symbiota in lower termites, and only bacteria in higher termites. **(B)** Caste and phenotype-associated transitions addressed through sociogenomic research. Left: non-reproductive or “apterous” (wingless) phenotypes of lower termites. Presoldiers and soldiers differentiate from workers in response to elevated juvenile hormone (JH) titers. Right: nymphs give rise to alates that become primary reproductives; a process akin to typical hemimetabolous insect development.

Termites are perhaps best know1n for their symbiotic associations with gut microbes (König et al., 2013; Brune, 2014) that are often linked to digestive processes, although lignocellulose digestion is not mediated entirely by gut microbes (Watanabe and Tokuda, 2010; **Figure 2A**). The more ancestral lower termites have tri-partite symbioses that include host, bacteria and protozoa; whereas in higher termites, symbiosis has been reduced to a two-way association between host and bacteria (but some higher termites also maintain ecto-symbiotic associations with fungi; Brune, 2014). The host component of termite symbiotic systems adds substantially to the digestive process both in terms of contributing enzymes and maintaining a favorable gut microenvironment for symbiosis and digestion to occur (Watanabe et al., 1998; Tartar et al., 2009; Scharf et al., 2011; Sethi et al., 2013a; Tokuda et al., 2014). Because of the high degree of interplay that occurs between the termite host and gut symbionts, a key idea moving forward will be to consider termites from the perspective of the “holobiont” (a single functional unit in which host and symbionts are physiologically tightly connected). Omic research has enabled a multifaceted systemic understanding of gut digestomes that is central to understanding the termite holobiome from an applied perspective (Scharf, 2015).

### Sociogenomics and Digestomics

The term *sociogenomics* was coined to describe the use of omic approaches for defining social life in molecular terms, which began with studies on the honey bee, *Apis mellifera* (Robinson et al., 2005). A parallel idea cited as rationale for many omic studies in social insects, including termites, is that solitary genes and traits were likely co-opted for new functions as solitary ancestors transitioned to social lifestyles (West-Eberhard, 2003; Nelson et al., 2007). Understanding such traits is essential for understanding termite social evolution (Miura and Scharf, 2011; **Figure 2B**). Another term used specifically in relation to digestive research is *digestomics*, which was coined to describe the collective pool of host and symbiont genes that collaborate to achieve high-efficiency lignocellulose digestion in the termite gut (Scharf and Tartar, 2008; Tartar et al., 2009; **Figure 2A**). Such terminology is useful because of the large number of symbionts that occupy termite guts and collaborate with the host in lignocellulose digestion. A related term is *termitosphere*, which is the full complement of gut and ectosymbiotic (nest) microbes present in termites, termite colonies, and their surrounding nest structures (Roose-Amsaleg et al., 2004; Bastien et al., 2013). Whether in relation to social, solitary or symbiont genes, proteins or other biomolecules, sociogenomic and digestomic research in termites has created an explosion of new sequence data.

## Omic Studies in Termites: *What has been Done?*

Based on a recent literature survey (**Table 1**), at the time of writing this article around 70 papers had been published describing omic efforts in termite systems. These studies include all the themes introduced above, as well as microbial 16S and 18S surveys.

### Taxonomic Distribution

In total, 82 termite species have been investigated using various omic approaches, with greater representation by lower than higher termites (72 vs. 28%). Among lower termites the top genera studied are important pest groups (*Reticulitermes* and *Coptotermes*), followed by non-pests from *Hodotermopsis*, *Mastotermes*, and *Cryptotermes*. Among higher termite genera, *Nasutitermes* dominate, followed by *Odontotermes*, *Trinervitermes*, and several other minor groups. Two termite genome sequences have now been published from the lower termite *Zootermopsis angusticollis* and the higher termite *Macrotermes natalensis* (see below).

### Host vs. Symbiont Investigation

Of the various omic studies to date considering symbiosis and symbiotic partnerships in termite systems, the majority have taken an exclusive symbiont-oriented approach (>60%), whereas a minority have considered the host termite separately (<20%). The remainder have considered host and symbiont together (∼20%). In the latter category of host and symbiont combined, some studies have been a case of “accidental metatranscriptomics” (because protist symbionts have polyadenylated transcripts that are represented in cDNA libraries along with host transcripts; e.g., Scharf et al., 2003, 2005; Steller et al., 2010), but others have been deliberate metatranscriptomic studies (e.g., Tartar et al., 2009; Raychoudhury et al., 2013; Sen et al., 2013). The greater emphasis on gut symbiota compared to the host termite is likely because of the stereotypically well-recognized presence of gut microbes in termites.

### Experimental Approaches and Types of Sequencing

In terms of experimental approaches taken, there has been an approximately equal split between descriptive and hypothesis-driven studies. Regarding the types of sequencing performed, transcriptomics and metatranscriptomics have been the dominant approaches (25 and 21% of studies), followed by microbial surveys for cataloging purposes (23%). The transcriptomic approaches used can be further divided into different methodologies such as cDNA library sequencing (Sanger, pyrosequencing or Illumina RNA-seq) and microarrays. Other efforts have targeted symbiont metagenomes (15%), symbiont or termite genomes (9%), proteomes (3%), and DNA methylomes (3%).

**Table 1:** A comprehensive literature summary of termite omic research, organized by approaches taken.

Omic approach taken	Termite group	Termite species	Host or symbiont	Tissue or fraction	Approach	Method	Major finding	Reference
Transcriptome	Lower	*Coptotermes formosanus*	Host	Whole-body polyphenic library	Hypothesis-driven (virgin vs. egg-laying queens)	Sanger sequencing + microarray	7663 ESTs sequenced that aligned into 4726 contigs; microarray analysis revealed 94 differentially expressed genes between virgin and reproductive queens	Husseneder et al. (2012)
			Combined	Whole workers	Hypothesis-driven	Sanger sequencing	1511 total unigenes (362 contigs + 1149 singletons)	Hussain et al. (2013)
		*Coptotermes gestroi*	Host	Head tissue	Descriptive	Sanger sequencing	3003 high quality ESTs were obtained that aligned into 695 unigenes (245 contigs and 450 singlets)	Do et al. (2014)
		*Cryptotermes secundus*	Combined	Whole workers and neotenic reproductives	Hypothesis-driven	Representational difference analysis, Sanger sequencing	187 differentially expressed library clones were identified that aligned into 35 unigene contigs	Weil et al. (2007)
		*Hodotermopsis sjostedti*	Host	Head tissue of differentiated workers and soldiers	Hypothesis-driven	Differential display, Sanger sequencing	11 candidate bands were identified, including the *SOL1* gene, which was detected mostly in soldier heads	Miura et al. (1999)
			Host	Mandibular tissue of workers, presoldiers, and soldiers	Hypothesis-driven (JHA induced gene expression)	Fluorescent differential display (FDD), Sanger sequencing	81 candidate bands identified by FDD that aligned into 12 unigenes upregulated in mandibular tissue during soldier differentiation	Koshikawa et al. (2005)
			Host	Whole worker termites without guts	Hypothesis-driven (JHA up and downregulated genes)	FDD, Sanger sequencing	28 candidate bands identified by FDD; 18 aligned into ca. 5 unigenes	Cornette et al. (2006)
			Host	Worker brain and subesophageal ganglion	Hypothesis-driven (JHA induced gene expression)	FDD, Sanger sequencing	No differences in expression patterns detected between pseudergates and soldiers; five genes up-regulated in brain and/or SOG during differentiation	Ishikawa et al. (2010)
			Host	Whole worker termites without guts	Hypothesis-driven (JHA up and downregulated genes)	Subtractive libraries, filter arrays, and Sanger sequencing	87 and 64 JHA up- and downregulated clones identified	Cornette et al. (2013)
		*Mastotermes darwiniensis*	Host	Worker head, thorax and front legs	Hypothesis-driven (genes upregulated by *Metarhizium anisopliae* infection)	Subtractive cDNA library, Sanger sequencing	The number of differentially expressed clones was not specified	Thompson et al. (2003)
		*Reticulitermes flavipes*	Combined	Whole worker termites	Hypothesis-driven	Sanger sequencing	19 total unigenes (13 contigs + 6 singletons)	Gao et al. (2012)
								
		Multi-species: *C. secundus* and *Cryptotermes cynocephalus*	Combined	Whole workers and neotenic reproductives	Hypothesis-driven	Representational difference analysis, Sanger sequencing	16 differentially expressed genes were identified in *C. cynocephalus* with significant homology to genes identified previously in *C. secundus*	Weil et al. (2009)
	Lower and higher	Multi-species: *H. sjostedti, Reticulitermes speratus*, and *Nasutitermes takasagoensis*	Host	Whole-body minus gut	Hypothesis-driven	454 pyrosequencing	>1.2 million quality-filtered reads yielding >400 million bases for each of the three species. Caste transcriptomes compared by GO and orthology searches. Putative JH and caste differentiation genes annotated	Hayashi et al. (2013)
	Higher	*Macrotermes gilvus*	Symbiont	Fungal ectosymbiont (*Termitomyces*)	Descriptive	Subtractive cDNA library, Sanger sequencing	1,382 and 325 EST contigs were obtained for non-subtracted (lignocellulose fed) and subtracted (lignocellulose minus lab diet) cDNA libraries	Johjima et al. (2006)
		*N. takasagoensis*	Host	Soldier frontal gland	Descriptive	454 pyrosequencing	50,290 sequence reads were assembled into 1111 contigs (774 unigenes)	Hojo et al. (2012)
		*Odontotermes formosanus*	Host	Head tissue	Descriptive	Illumina sequencing	116,885 unigene sequences; 30,646 with significant identity	Huang et al. (2012)
		Species unknown	Symbiont	Fungal ectosymbiont of higher termite (*Termitomyces albuminosus* )	Descriptive	454 pyrosequencing	6494 candidate genes (3301 contigs + 3193 singletons)	Yang et al. (2012)
Proteome	Lower	*R. flavipes (santonensis)*	Symbiont	Gut symbiota (bacteria, protist)	Descriptive	LC-MS/MS (ion trap) and 2-D PAGE	Tubulins proved to be the most suitable protein family with which to identify flagellate populations from hindgut samples	Bauwens et al. (2013)
								
	Higher	*Nasutitermes corniger*	Symbiont	Bacterial gut symbiota	Descriptive	LC-MS	886 proteins identified, 197 with known enzymatic function; very few cellulases identified	Burnum et al. (2011)
	Higher and lower	Multi-species: 12 species (10 lower, 2 higher)	Host	Labial glands of workers and soldiers	Descriptive	N-terminal peptide sequencing (Edman degradation)	Endogenous (host) endoglucanase cellulases were identified in worker labial glands of all species	Sillam-Dussès et al. (2012)
Metatranscriptome, metagenome, and 16S pyrosequencing	Higher	Multi-species: *Amitermes wheeleri, N. corniger*	Symbiont	Bacterial gut symbiota	Hypothesis-driven (differences between wood and dung feeders)	454 pyrosequencing	Firmicutes and Spirochaetes dominated in *A. wheeleri*, while Spirochaetes and Fibrobacteres dominated in *N. corniger*	He et al. (2013)
Metatranscriptome and proteome	Lower	*R. flavipes*	Combined	Worker termite gut and protist microbiota	Hypothesis-driven (comparison of cellulose vs. wood vs. lignin feeding)	454 pyrosequencing + LC-MS proteomics	347,798 sequence reads aligned into 97,254 singlets + 9553 differentially expressed contigs; proteome and transcriptome results showed congruence	Sethi et al. (2013a)
Metatranscriptome and proteome	Lower	*R. speratus*	Symbiont	Protist gut symbiota (hindgut lumen)	Descriptive	Sanger sequencing + proteomics	910 total clones sequenced; 580 candidate genes identified	Todaka et al. (2007)
Metatranscriptome	Lower	*C. formosanus*	Combined	Whole workers, nymphs, soldiers, and alates	Descriptive	Sanger sequencing (normalized polyphenic library)	25,939 candidate genes (16 691 contigs and 9248 singletons)	Zhang et al. (2012)
			Symbiont	Protist gut symbiota	Descriptive	454 pyrosequencing	75,122 candidate genes (2891 contigs + 72,231 singletons)	Xie et al. (2012)
		*H. sjostedti*	Combined	Worker termite gut (salivary gland, foregut, midgut, and hindgut) and protist microbiota	Descriptive	Sanger sequencing	Different compositions of expressed genes were identified across gut regions	Yuki et al. (2008)
		Multi-species: *R. speratus, H. sjostedti, Neotermes koshunensis, M. darwiniensis, Cryptocercus punctulatus*	Symbiont	Protist gut symbiota (hindgut lumen)	Hypothesis-driven	Sanger sequencing	910, 920, 1056, 1021, and 868 clones sequenced from each taxon (*n* = 4775) that aligned into 3780 unigene contigs; 77 full-length GHF7 cellulases were identified	Todaka et al. (2010)
								
		*R. flavipes*	Combined	Whole-body polyphenic library (non-normalized)	Descriptive	Sanger sequencing (random clones)	88 random clones were sequenced that aligned into 49 unigene contigs	Wu-Scharf et al. (2003)
			Combined	Whole-body polyphenic library (non-normalized)	Hypothesis-driven (worker vs. soldier)	Filter arrays, Sanger sequencing	105 differentially expressed clones were identified that aligned into 34 unigene contigs	Scharf et al. (2003)
			Combined	Whole-body polyphenic library (non-normalized)	Hypothesis-driven (worker vs. immature reproductive)	Filter arrays, Sanger sequencing	68 differentially expressed clones were identified that aligned into 25 unigene contigs	Scharf et al. (2005)
			Combined	Worker termite gut and protist microbiota	Hypothesis-driven	Sanger sequencing	6555 total transcripts (3044 host, 3511 protist symbiont)	Tartar et al. (2009)
			Combined	Whole-body polyphenic library (soldier, worker, alate, early and late larvae)	Hypothesis-driven (comparisons among castes)	Sanger sequencing (random clones)	15,259 random clones sequenced representing 6991 total genes	Steller et al. (2010)
			Combined	Worker termite gut and protist microbiota	Hypothesis-driven (comparison of JH, soldier head extract, live soldiers, and reproductives)	Microarray	543 total gut genes differentially expressed after 24-h exposures (151 host + 392 protist symbiont)	Sen et al. (2013)
			Combined	Worker termite gut and protist microbiota	Hypothesis-driven (comparison of wood and cellulose/paper feeding)	Microarray	544 total gut genes differentially expressed after 7-days feeding periods (236 host + 301 protist symbiont)	Raychoudhury et al. (2013)
		*Zootermopsis angusticollis*	Symbiont	Bacterial gut symbiota *(Treponema spirochetes*)	Hypothesis-driven (comparison of two *Treponema* in co-culture)	Illumina sequencing	Total database size = 3,855,671 reads; 45% of reads were 16S and 23S rRNAs; >97% of all non-rRNA genes were unique	Rosenthal et al. (2011)
Metagenome and proteome	Higher	*N. corniger*	Symbiont	Bacterial gut symbiota (P3 luminal contents)	Descriptive	Sanger sequencing + 454 pyrosequencing + LC-MS proteomics	12 bacterial phyla and 216 phylotypes identified; >71 Mb of DNA sequenced; ∼700 glycoside hydrolase domains corresponding to 45 different carbohydrate active enzymes were identified (including putative cellulases and hemicellulases)	Warnecke et al. (2007)
Metagenome and 16S survey	Higher	*Odontotermes yunnanensis*	Symbiont	Bacterial gut symbiota	Descriptive	454 pyrosequencing	548,807 total sequence reads; no evidence of lignases; 205 total cellulase and hemicellulase genes annotated	Liu et al. (2013)
Metagenome	LOWER	*C. gestroi*	Symbiont	Bacterial gut symbiota	Descriptive	Illumina *de novo* genome sequencing	316 candidate cellulase ORFs, 259 candidate hemicellulase ORFs, and 12 candidate pectinase ORFs	Do et al. (2014)
								
		*R. flavipes (santonensis)*	Symbiont	Bacterial gut symbiota	Descriptive	Functional screening (beta glucosidase) + Sanger sequencing	9 beta glucosidase positive clones were identified from GH1, GH3, and GH4	Mattéotti et al. (2011b)
			Symbiont	Bacterial gut symbiota	Descriptive	Functional screening (beta glucosidase) + Sanger sequencing	1 beta glucosidase positive clone was identified (GH1)	Mattéotti et al. (2011a)
			Symbiont	Bacterial gut symbiota	Descriptive	Functional screening (xylosidase) + Sanger sequencing	1 putative endo-1,4-beta-xylanase was identified from GH11	Mattéotti et al. (2012)
	Higher	*Globitermes sulphureus*	Symbiont	Bacterial gut symbiota	Descriptive	Functional screening (beta glucosidase) + Sanger sequencing	1 beta glucosidase positive clones was identified and functionally expressed	Wang et al. (2012)
		*Macrotermes annandalei*	Symbiont	Bacterial gut symbiota	Descriptive	454 pyrosequencing (bacterial fosmid libraries grown under selective conditions)	13 positive clones identified encoding 1 xylanase and 12 beta-glucosidases	Liu et al. (2011)
		*Microcerotermes* sp.	Symbiont	Bacterial gut symbiota	Descriptive	Functional screening (cellulase and xylanase) + Sanger sequencing	Fourteen independent active clones (2 cellulases and 12 xylanases) were obtained by functional screening (GHF 5,8,10,11)	Nimchua et al. (2012)
		*Pseudacanthotermes militaris*	Symbiont	Gut and fungal comb bacteria	Descriptive	454 pyrosequencing	1.46 Mbp of metagenome sequence	Bastien et al. (2013)
		*Trinervitermes trinervoides*	Symbiont	Bacterial gut symbiota	Descriptive	Functional screening (esterase)+ Sanger sequencing	68 fosmid clones were identified with esterase activity, of which the 14 most active were sub cloned and sequenced	Rashamuse et al. (2012)
			Symbiont	Bacterial gut symbiota	Descriptive	Functional screening (feruloyl “FAE” esterase) + Sanger sequencing	Seven FAE-positive fosmid clones were identified	Rashamuse et al. (2014)
Metabolome	Lower	*H. sjostedti*	Combined	Worker termite gut	Descriptive	Isotope-ratio mass spectrometry (IR-MS)	Localized the majority of glucose release from 13C-cellulose to the foregut region	Tokuda et al. (2014)
		*Z. angusticollis*	Combined	Worker termite gut	Descriptive	TMAH thermocemical lysis coupled with GC-MS	Results transformed the view of lignin degradation in the termite gut	Geib et al. (2008)
		*C. formosanus*	Combined	Worker termite gut	Descriptive	TMAH thermocemical lysis coupled with CP-MAS-NMR spectroscopy, and Py-GC/MS	During gut passage the native lignin macromolecular assembly undergoes structural modification but with conservation of the abundant β-*O*-4′ interunit lignin linkage and retention of the original aromatic properties	Ke et al. (2011)
		*C. formosanus*	Combined	Worker termite gut	Descriptive	TMAH thermocemical lysis coupled with GC-MS	Results suggest that the plant cell wall deconstruction process in *C. formosanus* consists of stepwise unlocking reactions that affect the lignin matrix and lignin–carbohydrate associations	Ke et al. (2013)
	Higher And lower	Multi-species: eight species (seven lower, one higher)	Host	Labial glands of workers and soldiers	Descriptive	HPLC MALDI-TOF and GC-TOF-MS	Hydroquinone and other glucose and benzene-linked compounds identified in labial gland secretions of workers and soldiers	Sillam-Dussès et al. (2012)
Genome	Lower	*C. formosanus*	Symbiont	Bacteroidales endosymbiont (phylotype CfPt1-2) of the cellulolytic protist *Pseudotrichonympha grassii*	Descriptive	Combination of Sanger and 454 pyrosequencing	1,114,206 bp chromosome containing 758 putative protein-coding sequences, 38 transfer RNA genes, and 4 rRNA genes	Hongoh et al. (2008a)
		*M. darwiniensis*	Symbiont	Blattabacterium bacterial endosymbiont	Descriptive	Illumina sequencing	594 candidate genes identified (544 protein-coding + 40 RNA-coding)	Sabree et al. (2012)
		*R. flavipes*	Symbiont	Bacterial gut symbiont (*Opitutaceae* bacterium strain TAV1; *Verrucomicrobia)*	Descriptive	Combination of Illumina + 454 pyrosequencing	Genome contains 6,051 genes with 5,987 CDS; 64 structural RNAs were identified with the presence of one rRNA operon	Isanapong et al. (2012)
		*Reticulitermes lucifugus*	Symbiont	Bacterial gut symbiont *(Sebadella termitidis* strain NCTC 11300^T^*; Phylum Fusobacteria)*	Descriptive	Combined Sanger, Illumina, and 454 pyrosequencing	4,486,650 bp long genome containing 4,264 predicted genes (4,210 protein-coding, 54 RNAs)	Harmon-Smith et al. (2010)
								
		*R. speratus*	Symbiont	Endomicrobia “TG-1” endosymbiont (phylotype Rs-D17) of the cellulolytic protist *Trichonympha agilis*	Descriptive	Combination of Sanger and 454 pyrosequencing	1,125,857 bp chromosome encoding 761 putative protein-coding genes	Hongoh et al. (2008b)
Genome, transcriptome, and DNA methylome	Lower	*Zootermopsis nevadensis*	Host	Worker, soldier, reproductive, larvae	Descriptive	Illumina + 454 pyrosequencing	562 Mb genome sequenced with 98x coverage; 96 miRNA, and 17,737 protein coding genes were identified	Terrapon et al. (2014)
Genome, fungal symbiont genome, and gut microbial metagenome	Higher	*Macrotermes natalensis* (and *Termitomyces* sp. symbiont)	Combined	Genomic DNA of *M. natalensis* queen and *Termitomyces* homokaryon, metagenomic DNA of major worker, minor soldier, and queen gut	Descriptive	Illumina sequencing	First sequencing of a tripartite symbiotic system; 1.3 Gb host genome, 84 Mb fungal symbiont genome; 816 Mb gut prokaryotic metagenomes; major emphasis on cellulose digestion; greatly reduced gut microbiome in queens relative to major workers and minor soldiers	Poulsen et al. (2014)
DNA methylome	Lower	*Coptotermes lacteus*	Host	Workers, soldiers and nymphs	Descriptive	Methylation-targeted amplification fragment length polymorphism (AFLP)	Found evidence for DNA methylation, but no differences in methylation levels among castes	Lo et al. (2012)
	Lower and higher	Multi-species: *H. sjostedti, R. speratus* and *N. takasagoensis*	Host	Whole-body minus gut	Hypothesis-driven	454 Pyrosequencing	>1.2 million filtered reads yielding >400 million bases for each of the three species. DNA methyltransferases putatively responsible for DNA methylation were represented in all three species	Hayashi et al. (2013)
	Lower	Multi-species: *R. flavipes, C. formosanus*	Host	Whole-body polyphenic libraries	Descriptive	Sanger sequencing	Signatures of high DNA methylation levels exist in *R. flavipes* and *C. formosanus.* Results suggest the presence of DNA methylation in R. flavipes and C. formosanus potentially at high levels or widely targeted across the lengths of genes, relative to other insect taxa	Glastad et al. (2013)
18S sequencing	Lower	Multi-species: *Reticulitermes, Zootermopsis, Cryptocercus*	Symbiont	Protist gut symbiota	Descriptive	454 pyrosequencing	Protist diversity estimated by 18S SSU sequencing is much higher than when estimated by protist morphology	Tai and Keeling (2013)
								
	Lower	*Z. angusticollis*	Symbiont	Protist gut symbiota (single cell)	Descriptive	Sanger sequencing	Seven protists identified by rRNA sequence	Tai et al. (2013)
18S and bacterial 16S sequencing	Lower	Multi-species: 24 lower termites and three *Cryptocercus* cockroaches	Symbiont	Protist and bacterial gut symbiota	Hypothesis-driven	454 pyrosequencing	Although microbial communities are vertically inherited and codiversification with the host termite has had a prominent role in structuring symbiont communities, dispersal appears to have a larger role in community composition	Tai et al. (2015)
16S sequencing	Lower	*C. formosanus*	Symbiont (positions 27-1492)	Cuticular bacteria	Hypothesis-driven	Sanger sequencing	25 total ribotypes detected (20 and 14 from simple and extended families)	Husseneder et al. (2010b)
			Symbiont (positions 27-1492)	Bacterial gut symbiota (whole gut)	Hypothesis-driven	Sanger sequencing	1,876 total 16S reads that sorted into 213 bacteria ribotypes and 13 phyla	Husseneder et al. (2010a)
		Multi-species: *R. flavipes (santonensis), H. sjostedti, Z. nevadensis, M. darwiniensis, Kalotermes flavicollis, Neotermes castaneus, C. secundus*	Symbiont (positions 27-1492)	Bacterial endosymbionts of protist gut symbionts	Descriptive	Sanger sequencing	Each protist morphotype harbored “Endomicrobia” from unique phylogenetic lineages	Stingl et al. (2005)
		Multi-species: *R. flavipes, C. formosanus, Z. angusticollis*	Symbiont (positions 63-1492)	Spirochaete gut symbiota (whole gut)	Descriptive	Sanger sequencing	>21 new species of *Treponema* identified in each of the three species studied	Lilburn et al. (1999)
		*R. flavipes*	Symbiont (entire SSU region)	Bacterial gut symbiota (hindgut lumen)	Descriptive	Sanger sequencing + ARDRA analysis	Six phyla and 261 species-level phylotypes estimated	Fisher et al. (2007)
			Symbiont (V5–V6 region)	Bacterial gut symbiota (hindgut lumen)	Hypothesis-driven	454 pyrosequencing	475,980 total 16S reads that sort into eight major bacterial phyla and 4761 species-level phylotypes (5% divergence level)	Boucias et al. (2013)
		*R. flavipes (santonensis)*	Symbiont positions (27-1492)	Bacterial gut symbiota (midgut, protozoa, hindgut fluid and wall)	Descriptive	Sanger sequencing + T-RFLP analysis	392 clones sequenced; seven major phyla and >200 species-level bacterial ribotypes identified	Yang et al. (2005)
								
		*R. speratus*	Symbiont (positions 563-1114)	Bacterial gut symbiota (whole gut)	Descriptive	Sanger sequencing	1344 clones sequenced; 11 phyla and 268 species-level phylotypes identified	Hongoh et al. (2003)
		*Z. angusticollis*	Symbiont (positions 27-1492)	Bacterial gut symbiota	Hypothesis-driven (effects of antibiotic rifampicin)	Sanger sequencing	Six and 17 species-level OTUs were identified for rifampin and control treatments (*n* = 85–87 clones)	Rosengaus et al. (2011)
	Higher	*Cornitermes cumulans*	Symbiont (positions 21 or 27-907)	Whole gut	Descriptive	Sanger sequencing	>8 Phyla identified (species-level estimates not provided)	Grieco et al. (2013)
		*Cubitermes niokoloensis*	Symbiont (positions 338-518)	Bacterial symbionts from gut regions, soil and mound	Hypothesis-driven	Sanger sequencing + DGGE	212 total clones sequenced; 101 different species-level phylotypes identified	Fall et al. (2007)
		Multi-species: *Odontotermes somaliensis*, *Odontotermes* sp., *Microtermes* sp.	Symbiont (positions 27-1492)	Bacterial gut symbiota	Hypothesis-driven	Sanger sequencing	100, 100 and 96 clones sequenced from each taxon; 151 different phylotypes identified	Makonde et al. (2013)
		Multi-species:	Symbiont (positions 341-806)	Bacterial gut symbiota	Descriptive	454 pyrosequencing	Performed 16S sequencing on nine fungus-growing termite species from one geographic region of Ivory Coast; Identified 16 phyla and 42 genera total, with 11 genera occurring in all nine species	Otani et al. (2014)
		*N. corniger*	Symbiont (V1–V2 and V8 regions)	Bacterial gut symbiota (P3 lumen)	Hypothesis-driven	454 pyrosequencing	2269 species-level OTUs of which 1617 and 652 were from the V1–V2 and V8 regions, respectively	Engelbrektson et al. (2010)
			Symbiont (V3–V4 region)	Bacterial gut symbiota (six whole gut regions)	Descriptive	454 pyrosequencing	3,200-26,000 16S reads per gut region (crop, midgut and paunch P1–P4) that sort into seven major bacterial phyla	Köhler et al. (2012)
		*N. takasagoensis*	Symbiont (positions 27-1390)	Bacterial gut symbiota (whole gut)	Hypothesis-driven	Sanger sequencing + T-RFLP analysis	388 total clones sequenced; 10 major phyla identified; 31–43 species-level phylotypes	Miyata et al. (2007)
		*T. trinervoides*	Symbiont (positions 1170-1492)	Bacterial gut symbiota	Hypothesis-driven (difference between grass and sugarcane feeding field colonies)	454 pyrosequencing	2274 and 2943 species-level OTUs sampled from sugarcane and grass feeding colonies (1% divergence level); nine major phyla sampled; Firmicutes and Bacteroidetes most common	Sanyika et al. (2012)

## Omic Studies in Termites: *What has been Revealed?*

### Genomics

#### Host Termite Genomes

At present only two termite genome sequences are available (**Table 1**); one from the lower termite *Zootermopsis nevadensis* (Terrapon et al., 2014) and one from the higher termite *M. natalensis* (Poulsen et al., 2014). *Z. nevadensis* was selected for sequencing based on its small genome size of 562 Mb relative to other termites, most of which are over 1000 Mb (Koshikawa et al., 2008). The *Z. nevadensis* sequencing approach involved shotgun genome sequencing of genomic DNA from symbiont-free soldier heads (*n* = 50 and 150 heads for 2 and 20 kb libraries, respectively). The transcriptomes of castes and various phenotypes were also sequenced for both gene prediction and comparative transcriptomic purposes. Transcriptome data were also used to search for DNA methylation machinery and methylation/epigenetic differences among castes and developmental stages.

The *Z. nevadensis* genome provided the first hints into how termites differ at the genome level from their eusocial counterparts in the order Hymenoptera, which evolved sociality independently. For making socio-evolutionary comparisons, emphasis was placed on gene family expansions, male fertility, chemoreception, immunity, polyphenism/division of labor, and potential epigenetic caste regulation. An expansion of genes related to male fertility and upregulated gene expression in male reproductives are consistent with differences in mating biology between termites and Hymenoptera. Regarding chemoreception, divergent numbers of genes and gene families relative to Hymenoptera were identified, as were variations in chemoreception gene expression among castes. Regarding caste polyphenism and division of labor, caste-associated gene expression profiles were readily identifiable. Key caste-regulatory and reproduction-associated genes identified through preceding work (e.g., hexamerins, vitellogenins, and CYP genes) were further defined and verified as gene families at the genomic level. Interestingly, there are 76 cytochrome P450 genes in the *Z. nevadensis* genome; which is nearly 2x as many as encoded by the honey bee genome (Honey Bee Genome Sequencing Consortium, 2006). Lastly, DNA methylation signatures and patterns of alternative splicing provided some evidence to suggest epigenetic caste regulation (see later).

The *M. natalensis* sequencing considered not only the host genome, but also the entire tri-partite system of this higher fungus-growing termite. This included the 1.3 Gb host genome, the 84 Mb genome of the *Termitomyces* sp. fungal symbiont and 816 Mb of prokaryotic gut metagenome from major workers, minor soldiers, and queens. Emphasis was placed mostly on cellulose digestion, which revealed a rich complement of glycosyl hydrolases from host, fungi, and gut microbes that likely collaborate in lignocellulose digestion. Another major finding was that gut microbiota composition is reduced by over 50% in queens relative to workers and soldiers, suggesting that queen gut microbiota undergo substantial compositional changes during colony founding, which points toward the local environment or other external factors as sources of microbiota as incipient colonies grow and age. Moving forward, the *Z. nevadensis* and *M. natalensis* genomes will be important resources for termitologists, and will also provide important scaffolds for assembly of additional termite genomes that will facilitate study of genes related to many evolutionary and biological processes.

#### Individual Symbiont Genomes

Five individual symbiont genomes have been sequenced (**Table 1**), with several others published or in progress since the writing of this article. No protist genomes have yet been sequenced. Two bacterial endosymbionts of hindgut protists from *Coptotermes formosanus* and *Reticulitermes speratus* (phylum Elusimicrobia or “TG1”) were the first symbiont genomes sequenced; they were obtained from isolated individual cells after whole-genome amplification (Hongoh et al., 2008a,b). No lignocellulase genes were identified; however, both genomes encoded capabilities to fix nitrogen, recycle host nitrogen wastes for amino acid and cofactor biosynthesis, and import glucose and xylose as energy and carbon sources. The next symbiont genomes were from gut bacteria in the phyla Verrucomicrobia and Fusobacteria, from the termites *Reticulitermes flavipes* and *R. lucifugus* (Harmon-Smith et al., 2010; Isanapong et al., 2012). These genomes were from culturable isolates and were found to encode genes related to cellulose degradation and nitrogen fixation. Another example is the genome of an obligate fat body endosymbiont *Blattabacterium* from the basal termite *Mastotermes darwiniensis* (Sabree et al., 2012). This bacterium displays a reduction in genome size and loss of genes required for amino acid production relative to free-living gut bacteria, which is consistent with its ability to recycle nitrogenous wastes and its role as a co-evolved endosymbiotic partner of the host termite.

#### Symbiont Metagenomes

At the time of writing this article, at least 12 prokaryotic metagenomes had been partially sequenced (**Table 1**). Most metagenome publications have reported on lignocellulase identification from genome sequences of gut bacteria that selectively grew on lignocellulose media (Liu et al., 2011; Mattéotti et al., 2011a,b, 2012; Nimchua et al., 2012; Rashamuse et al., 2012, 2014; Wang et al., 2012). Another study used targeted xylanase screening from gut and ectosymbiotic fungi-associated bacteria of the higher termite *Pseudacanthotermes militaris* (Bastien et al., 2013). Other studies took broader approaches to sequence from gut bacterial communities of higher termites. By combining metagenome sequencing with 16S surveys and metatranscriptomics, these studies revealed new information on bacterial cellulase diversity from termites with different symbiosis strategies (i.e., with and without fungal ectosymbionts; Warnecke et al., 2007; Liu et al., 2013) and from different feeding guilds (dung vs. wood; He et al., 2013). While these studies provided a wealth of new high-impact information on bacterial symbionts, they did not consider how symbionts from the gut and/or nest termitosphere collaborate with or complement the host termite.

### Transcriptomics

#### Host Transcriptome

Around 15 transcriptomic studies to date have focused on physiological processes or tissues in the host termite (**Table 1**). Early studies looked for caste-biased gene expression, but the approaches employed had low resolving power and typically revealed only small numbers of differentially expressed genes. These studies mainly used subtractive hybridizations or cDNA “macro” arrays (reviewed by Miura and Scharf, 2011). Also, these early studies in lower termites often fell into the category of “accidental metatranscriptomics” as described earlier. The majority of focus in termite transcriptomic work has been on differences among castes or during caste differentiation (reviewed by Miura and Scharf, 2011). Mainly, newer studies are considered here.

Because of the importance of juvenile hormone (JH) to soldier caste differentiation and the reliability of JH treatment for inducing soldier caste differentiation, continuing focus has been placed on this transition in hypothesis-driven studies that combine JH assays with transcriptomics (e.g., Cornette et al., 2013; Sen et al., 2013). Caste-regulatory primer pheromones and the social environment have also been studied in the same context (Tarver et al., 2010; Sen et al., 2013). Other studies have included tissue-directed subtractive hybridizations, random/*de novo* cDNA library sequencing and/or cDNA oligonucleotide microarrays to reveal caste-biased gene expression (Weil et al., 2009; Ishikawa et al., 2010; Leonardo et al., 2011; Hojo et al., 2012; Huang et al., 2012; Husseneder et al., 2012; Terrapon et al., 2014). The over-arching themes emerging from this work include caste and morphogenesis-associated gene expression, endocrine signaling, vitellogenesis, reproduction-related processes, and regulatory mechanisms that maintain juvenile worker states in lower termites.

The immune response is another aspect of host termite physiology investigated through transcriptomics. Four studies have revealed responses to immune challenges by both stereotypical and unprecedented immune-responsive genes (Thompson et al., 2003; Yuki et al., 2008; Gao et al., 2012; Hussain et al., 2013). Finally, an emerging theme has been to investigate pathogen-xenobiotic interactions at the transcriptome level (Husseneder and Simms, 2014; Sen et al., 2015).

#### Symbiont-Host Metatranscriptomes

In addition to host-targeted studies noted above, other studies have considered symbiont or host-symbiont metatranscriptome composition (**Table 1**). Early examples in this category showed worker-biased expression of protist cellulases (Scharf et al., 2003) and differential expression of symbiont cellulases between dispersing and non-dispersing adult reproductives (Scharf et al., 2005). Subsequent studies focused on metatranscriptome composition of bacteria, protist and/or fungal symbionts, mostly for the purpose of identifying digestive cellulases (reviewed by Scharf and Tartar, 2008). Recent work has probed deeper into gut metatranscriptomes by taking advantage of both traditional and next-generation sequencing technology (Todaka et al., 2010; Rosenthal et al., 2011; Xie et al., 2012; Zhang et al., 2012; He et al., 2013). Other work has sought to partition host and symbiont digestive contributions and identify candidate enzymes expressed specifically in response to wood (i.e., complex lignocellulose), cellulose and lignin feeding (Tartar et al., 2009; Raychoudhury et al., 2013; Sethi et al., 2013a).

One microarray study investigated gut metatranscriptome changes in responses to JH, primer pheromones and socio-environmental conditions, suggesting interesting linkages between gut symbiota and caste differentiation (Sen et al., 2013). Another microarray study investigated host and symbiont gene expression in response to pathogen and nicotinoid-insecticide challenges, providing new insights into immunological roles played by bacterial and protist gut symbionts in defending against invading fungal and bacterial pathogens (Sen et al., 2015), building on the ideas of extended disease resistance as conferred by fecal nest bacteria (Chouvenc et al., 2013) and gut microbiota (Rosengaus et al., 2014).

### Proteomics

Proteomics (**Table 1**) is important to validate transcriptome studies, particularly for determining if a gene’s presence and/or its transcription and translation are proportional. For example, proteomic studies in a higher termite were unable to identify most of the bacterial cellulase proteins predicted by metagenome sequencing (Warnecke et al., 2007; Burnum et al., 2011). Alternatively, proteomic studies in lower termites were able to identify both protist cellulases and other host lignocellulases initially identified via metatranscriptome sequencing (Todaka et al., 2007; Sethi et al., 2013a). Another study investigated proteins present in labial gland secretions of 12 lower and higher termite species, identifying endogenous GHF9 cellulases as dominant components of worker labial gland secretions in most species investigated (Sillam-Dussès et al., 2012). Another study used proteomics to catalog gut microbial communities, but with limited resolution (Bauwens et al., 2013). Clearly, more proteomic efforts are needed to resolve issues related to: (1) congruency between nucleic acid and protein sequencing approaches, and (2) to verify open reading frames predicted by metagenome and transcriptome sequencing.

### DNA Methylomes

Four studies to date have looked at methylation signatures across termite castes with somewhat differing results. A seminal study used a methylation-targeted amplification fragment length polymorphism (AFLP) approach in *Coptotermes lacteus* to look for methylation signature differences among castes (Lo et al., 2012). Evidence of methylation was found, but no significant caste-associated methylation patterns were identified.

A subsequent study was done *in silico* using database sequences from *R. flavipes* and *C. formosanus* (Glastad et al., 2013). In this study and the two described below, transcriptome data were mined to determine the specific distribution of CpG dinucleotides (i.e., 5′–3′ cytosine followed by guanine), in order to predict DNA methylation levels *in silico*. Evidence of DNA methylation machinery and methylation signatures was found at high levels among expressed genes. Results also suggested that DNA methylation in *R. flavipes* is targeted to genes with ubiquitous (rather than differential) expression among castes and morphs. A third study examined host transcriptomes of three termite species that included two lower (*Hodotermopsis sjostedti*, *R. speratus*) and one higher termite (*Nasutitermes takasagoensis*; Hayashi et al., 2013). Pyrosequencing was done in combination with 69 caste and phenotypic libraries from the three termite species. Sequence analysis revealed that DNA methyltransferases potentially responsible for DNA methylation were present in each species, and verified the presence of methylation signatures. However, only limited evidence of caste-associated methylation profiles was detectable across the three species.

Finally, DNA methylation was assessed in *Z. nevadensis* as part of genome and transcriptome sequencing efforts (Terrapon et al., 2014). Transcriptome data were used to determine the specific distribution of CpG dinucleotides, in order to make *in silico* predictions of DNA methylation levels and explore for epigenetic differences among castes. In addition to verifying the presence of genes that encode for DNA methylation machinery (i.e., DNA methyltransferases 1 and 3), results showed greater methylation of genes rather than intergenic DNA, and a greater presence in introns than exons. This evidence, along with findings that alternatively spliced genes have greater degrees of methylation, suggests intronic methylation may impact alternative splicing.

While it is clear that DNA methylation exists in termites, so-far inconclusive results have been obtained to suggest epigenetic caste regulation. As concluded previously in relation to genetic caste determination (Vargo and Husseneder, 2009), the field of epigenetic caste regulation is in its infancy and epigenetic phenomena may or may not be relevant in natural colonies. More importantly, *in silico* methylation studies can only suggest that methylation may exist and which genes might be differentially methylated. Functional/translational research will be required to verify whether or not such genes truly are methylated, as well as the functions of those genes.

### Metabolomics

Metabolomic studies are useful for assessing *in situ* processes, both as an exploratory approach and for functional/translational studies to verify nucleotide sequences. Soldier defensive secretions previously received much attention in this respect (Prestwich, 1984; Nelson et al., 2001). A more recent study investigated chemical components of labial gland secretions in soldier and worker termites from 7 lower and 1 higher termite (Sillam-Dussès et al., 2012). This study confirmed hydroquinone and other glucose and benzene-linked compounds as common labial gland secretions among most species.

Other metabolomic studies have focused on lignocellulose digestion. One main question addressed has been: *does lignin digestion or modification occur during passage through the termite gut*? Several studies over the past 25 years have addressed this question (reviewed by Ni and Tokuda, 2013) but recent metabolomic studies have been particularly informative (Geib et al., 2008; Ke et al., 2011, 2013). In general, findings are consistent regarding modification of lignin during passage through the gut, but evidence of actual lignin depolymerization has been more elusive. One possible reason for this could relate to insufficient detection procedures. Another possibility is that lignin-ether bonds, broken during depolymerization, only remain in this state for a short time and thus appear as intact lignin in frass. The induction of numerous antioxidant and detoxification enzymes by lignin feeding, as well as increased saccharification in the presence of lignin-associated phenoloxidases, supports the latter possibility (Sethi et al., 2013a). Despite convincing evidence of lignin modification during passage through the termite gut, and related omic studies revealing lignin-associated changes in host oxidative enzymatic machinery, the topic of lignin digestion/modification in termite guts remains contentious (Brune, 2014).

Another aspect of termite metabolomic research considers cellulose digestion and relative contributions of host and symbiont to this process. A recent metabolomic study investigated *in situ* digestion of ^13^C-labeled crystalline cellulose by *H. sjostedti* (Tokuda et al., 2014). Novel insights obtained related to both cellulose digestion and nitrogen metabolism. The results not only confirmed preceding work showing that endogenous cellulose digestion by the host is substantial, but also suggested other novel possibilities; for example (i) a significant digestive contribution by hindgut bacteria is phosphorolysis of cello-oligosaccharides to glucose-1-phosphate, and (ii) essential amino acid acquisition occurs via lysis of hindgut microbes obtained through proctodeal trophallaxis. The rapid buildup of glucose observed in the foregut agrees well with prior studies showing that host foregut cellulases can produce high levels of glucose directly from wood lignocellulose (Scharf et al., 2011; Sethi et al., 2013a,b). Additionally, higher glucose levels observed in the hindgut than other regions agrees with estimates that glucose release from lignocellulose is about 1/3 host and 2/3 symbiont (Scharf et al., 2011). However, since this study only focused on metabolite identification in gut tissue, it could not account for nutrients/metabolites transported out of the foregut and catabolized in other areas of the body.

### Symbiont 16S and 18S Surveys

Bacterial 16S rRNA sequence surveys have been used extensively for cataloging bacteria and archaea (Wang and Qian, 2009), whereas 18S small subunit (SSU) rRNA surveys are just beginning to gain attention for cataloging protist symbionts (Tai and Keeling, 2013). Over 20 bacterial 16S surveys have been published to date using both cloning-dependent and -independent, high- and low-throughput approaches (**Table 1**). Highly variable species-level compositions have been obtained across the different termite species investigated, but, in general, six major bacterial phyla are represented across higher and lower termites: Bacteroidetes, Firmicutes, Spirochaetes, Proteobacteria, Fibrobacteres, and Elusimicrobia (Brune, 2014). Surveys conducted in parallel with higher-termite metagenome studies have been very informative for matching functional and taxonomic diversity (Warnecke et al., 2007; He et al., 2013); however, a study comparing multiple colonies through pyrosequencing of 16S amplicons found that bacterial compositions were different among colonies and likely influenced by local environment (Boucias et al., 2013). Additionally, 16S surveys revealed that lignocellulosic diet shifts have no short-term impacts on termite and cockroach microbiota composition (Sanyika et al., 2012; Boucias et al., 2013; Schauer et al., 2014). Another 16S survey of fungus-growing termites suggested a core microbiota of 42 genera that was shared among all nine termite species tested (Otani et al., 2014). This core microbiota was very different from other higher and lower termites, leading the authors to conclude the 42 common genera represent a core microbiota of fungus-growing termites. Conversely, since the termites were sampled from a limited geographic area it is possible that the core genera represent common microbes acquired from the local environment.

In comparison to prokaryotic 16S surveys, comparatively few protist 18S SSU surveys have been conducted (**Table 1**). These studies, conducted using a combination of cloning-dependent and independent approaches, have been transformative. Two studies provided new evidence to suggest greater protist symbiont diversity than originally indicated by traditional morphological identification (James et al., 2013; Tai et al., 2013). Two other studies used high-throughput 16S and 18S SSU sequencing to compare 24 lower termites with three wood-feeding cockroaches (Tai and Keeling, 2013; Tai et al., 2015). Like their predecessors, these studies found protist diversity to be higher than when estimated by morphology, and also that protist symbiont taxa tend to be highly endemic to a host genus, which is different than relationships between termite hosts and bacterial symbiota. These findings illustrate the significant opportunities that exist for development of high-throughput techniques for assessing protist symbiont communities and studying protist-bacterial symbiont relationships.

## Needs and Opportunities

Termite omic research in the last 10–15 years has led to a new era of understanding for termite and symbiont biology. Omics has also enabled the development of new unparalleled resources (i.e., transcriptome, genome, proteome, metabolome, symbiont meta-omic, and symbiont rDNA) useful for moving ahead with targeted functional work. The stage is now set for making significant headway in many aspects of termite research, including, but not limited to digestion, symbiosis, caste differentiation, and social evolution. However, key needs and opportunities remain in specific areas that seem particularly relevant for filling in knowledge gaps and potentially leading to transformative, paradigm-shifting outcomes.

Having the *Z. nevadensis* and *M. natalensis* genomes available not only facilitates further study of genes related to a range of evolutionary and biological processes, but these resources also provide important scaffolds for assembly of additional lower and higher termite genomes. Once multiple termite genomes are available, this would certainly better inform our view of termite social evolution. On the topic of host-symbiont “hologenomes,” sequencing more host genomes and symbiont metagenomes from the same termites concurrently (as recently done for *M. natalensis*), would provide unprecedented insights into the scope of interactions and synergies occurring in termite holobiomes. Such efforts could further reveal important differences between clades of higher and lower termites, leading to new evolutionary insights. Such datasets would also provide unmatched resources for advancing integrative sociogenomic, digestomic, termitosphere, and other research topics.

On the topic of proteomics, more studies are needed in species that have had genomes, transcriptomes, metagenomes, or metatranscriptomes sequenced. Combining proteomics with nucleic acid sequencing will better resolve gene prediction models and better test for congruency between transcription and translation profiles. On the topic of metabolomics, termite digestion remains an area much in need of metabolomic research focusing on how complex lignocellulose is broken down in termite guts and converted to energy. Also, tracking metabolites as they leave the gut and are utilized in the termite body would be very informative for testing hypotheses on the relative importance of nutrient flow into symbiont metabolic pathways.

On the topic of DNA methylomics, while it is now clear that DNA methylation happens in termites, so-far inconclusive results have been obtained regarding the role of DNA methylation in caste regulation. *In silico* methylation studies as performed can only suggest that methylation may exist and which genes are potentially differentially methylated. Functional and translational research is needed to understand the roles of such genes.

Substantial opportunities and needs still remain for 16S and 18S rRNA-based symbiont cataloging. Protist 18S SSU cataloging capabilities in particular have recently been developed, and can continue to improve provided that several conditions are met, such as: (1) appropriate primers can be developed, (2) statistically sound sampling regimes can be developed at biologically relevant scales, (3) single-cell microbiology and other data sources can be integrated, and (4) appropriate analytical tools developed (Tai and Keeling, 2013). This line of research has already begun to transform the view of protist diversity and co-evolution with host termites but more studies are needed in different termite species with established omic resources.

Finally, regarding prokaryotic 16S surveys, much has already been done, but an important gap in knowledge is the extent to which environment influences bacterial microbiota composition. This is important information for understanding differences in behavior and physiology across the geographic range for a termite species, as well as potentially for limiting the extent to which generalizations can be made about the relative importance of individual microbes or core microbiota in gut communities.

## Conclusion

This review has covered many aspects related to outcomes, findings and trends resulting from termite omic research. To date, omic research in diverse termite species has provided key insights into caste differentiation, digestion, pathogen defense and microbiomes, and most recently has provided two termite genome sequences. Termite omics has also created important tools and resources for conducting targeted, functional, translational, and applied research. However, these resources have only received limited attention to date for asking hypothesis-driven questions to elucidate the functional and evolutionary significance for pools of identified genes, proteins, and microbes. In recent years sequencing has rapidly moved into the realm of super high-throughput, with accompanying assembly and analyses requiring proportional super-computing power and bioinformatics expertise, but only limited resolution of biology or function. Transitioning from research that produces lists of genes, proteins and microbes, to research that determines their functional significance, is where the most important challenges lie for the next phases of termite science.

## Conflict of Interest Statement

The author declares that the research was conducted in the absence of any commercial or financial relationships that could be construed as a potential conflict of interest.

## Footnotes

1 The singular term “omic" is used as an adjective in this review.

2 The plural term “omics" is used as a noun.
